# Smartphone-based activity measurements in patients with newly diagnosed bipolar disorder, unaffected relatives and control individuals

**DOI:** 10.1186/s40345-020-00195-0

**Published:** 2020-11-02

**Authors:** Sharleny Stanislaus, Maj Vinberg, Sigurd Melbye, Mads Frost, Jonas Busk, Jakob E. Bardram, Lars Vedel Kessing, Maria Faurholt-Jepsen

**Affiliations:** 1 The Copenhagen Affective Disorder Research Center (CADIC), Psychiatric Center Copenhagen, Department O, 6243, Rigshospitalet, Blegdamsvej 9, 2100 Copenhagen, Denmark; 2 Monsenso ApS, Langelinie Allé 47, Copenhagen, Denmark; 3 Copenhagen Center for Health Technology (CACHET), Department of Health Technology, Technical University of Denmark, Lyngby, Denmark

**Keywords:** Bipolar disorder, Smartphone, Remote monitoring, Activity, Electronic monitoring

## Abstract

**Background:**

In DSM-5 activity is a core criterion for diagnosing hypomania and mania. However, there are no guidelines for quantifying changes in activity. The objectives of the study were (1) to investigate daily smartphone-based self-reported and automatically-generated activity, respectively, against validated measurements of activity; (2) to validate daily smartphone-based self-reported activity and automatically-generated activity against each other; (3) to investigate differences in daily self-reported and automatically-generated smartphone-based activity between patients with bipolar disorder (BD), unaffected relatives (UR) and healthy control individuals (HC).

**Methods:**

A total of 203 patients with BD, 54 UR, and 109 HC were included. On a smartphone-based app, the participants daily reported their activity level on a scale from −3 to + 3. Additionally, participants owning an android smartphone provided automatically-generated data, including step counts, screen on/off logs, and call- and text-logs. Smartphone-based activity was validated against an activity questionnaire the International Physical Activity Questionnaire (IPAQ) and activity items on observer-based rating scales of depression using the Hamilton Depression Rating scale (HAMD), mania using Young Mania Rating scale (YMRS) and functioning using the Functional Assessment Short Test (FAST). In these analyses, we calculated averages of smartphone-based activity measurements reported in the period corresponding to the days assessed by the questionnaires and rating scales.

**Results:**

(1) Smartphone-based self-reported activity was a valid measure according to scores on the IPAQ and activity items on the HAMD and YMRS, and was associated with FAST scores, whereas the majority of automatically-generated smartphone-based activity measurements were not. (2) Daily smartphone-based self-reported and automatically-generated activity correlated with each other with nearly all measurements. (3) Patients with BD had decreased daily self-reported activity compared with HC. Patients with BD had decreased physical (number of steps) and social activity (more missed calls) but a longer call duration compared with HC. UR also had decreased physical activity compared with HC but did not differ on daily self-reported activity or social activity.

**Conclusion:**

Daily self-reported activity measured via smartphone represents overall activity and correlates with measurements of automatically generated smartphone-based activity. Detecting activity levels using smartphones may be clinically helpful in diagnosis and illness monitoring in patients with bipolar disorder.

*Trial registration* clinicaltrials.gov NCT02888262

## Background

Activity and energy level are core symptoms of bipolar disorder (BD) (Kupfer et al. 1974). Hypomanic and manic episodes are characterized by increased energy, enhanced engagement in social activities and increased psychomotor activity (Carlson and Goodwin 1973; Faurholt-Jepsen et al. 2015; Frye et al. 2009), whereas depressive episodes are often associated with loss of energy, withdrawal from social activities and psychomotor retardation or agitation (Lewinsohn and Graf 1973; Sobin and Sackeim 1997). Several studies have suggested that increased activity is a persistent and prominent symptom of (hypo)mania (Bauer et al. 1991; Benazzi 2007; Cheniaux et al. 2014), whereas decreased activity has been reported in patients with BD during depressive episodes and remission compared with healthy control individuals (HC) (Crescenzo et al. 2017; Faurholt-Jepsen et al. 2012; Scott et al. 2017). The relevance of increased activity in hypomania was recently stressed in the DSM-5 where elevated activity or energy is a mandatory core criterion of (hypo)mania in addition to elevated or irritable mood (DSM-5 2013) resulting in a substantial reduction in the prevalence of (hypo)manic episodes diagnosed with DSM-5 compared with DSM-IV (Fredskild et al. 2019).

Previous studies investigating activity and energy levels in patients with BD have primarily relied on observer-based ratings, self-reported questionnaires, and wrist-and thoracic worn accelerometers/heart rate sensors (Faurholt-Jepsen et al. 2012; Krane-Gartiser et al. 2014). Retrospective questionnaires are prone to recall bias (Stone et al. 2003), and self-reported physical activity is often over-estimated compared to objective measurements (Vancampfort et al. 2016). Further, wrist-worn accelerometer relies on only one parameter. Smartphones can collect subjective as well as objective measurements of activity relatively unobtrusively and during naturalistic settings and provides a platform where several parameters reflecting activity can be combined. Several studies have found smartphone-based self-reports of activity feasible to collect daily smartphone-based recordings in real-time in patients with BD (Faurholt-Jepsen et al. 2015; Matthews et al. 2016; Tsanas et al. 2017). Similarly, automatically generated smartphone-based data might capture changes in activity within speech, mobility and social interaction in patients with BD (Faurholt-Jepsen et al. 2015; Beiwinkel et al. 2016; Grünerbl et al. 2012; Palmius et al. 2017; Rohani et al. 2018). Our group recently found that by applying advanced machine learning algorithms to analyze automatically generated smartphone-based data, including screen features and call- and text logs, it was possible to discriminate between patients with BD and HC. The findings suggest that smartphone-based automatically generated data may represent a potential diagnostic marker for bipolar disorder that in future may be clinically useful (Faurholt-Jepsen et al. 2019). Nevertheless, the validity of daily self-reported and automatically generated smartphone-based activity has not been systematically validated. Further, it has not been investigated whether daily self-reported and automatically generated smartphone-based activity differs between patients with newly diagnosed BD, unaffected first-generation relatives (UR), and HC. In this study, all patients with BD were included independently of their mood state.

## Aims of the study

The present study had three aims:

Firstly, to investigate daily smartphone-based self-reported and automatically generated activity, respectively, against validated measurements of activity including (1) a validated questionnaire for physical activity, (2) activity assessed by trained clinicians according to activity items on validated rating scales of severity of depression and mania, respectively, (3) functioning according to clinical assessment with a validated rating system.

Secondly, to investigate daily smartphone-based self-reported activity and automatically generated activity against each other.

Thirdly, to investigate differences in daily self-reported and automatically generated smartphone-based activity in patients with newly diagnosed BD, UR, and HC.

We hypothesized that (1) daily smartphone-based self-reported and automatically generated activity represents valid measurements of activity according to questionnaires and clinical ratings of activity (2) daily smartphone-based self-reported and automatically generated activity are associated, and (3) activity level is decreased for patients with newly diagnosed BD compared with HC individuals and intermediary for UR individuals.

## Methods

### Study design

The present study is part of the larger ongoing Bipolar Illness Onset studies (BIO study) (Kessing et al. 2017). Three groups of participants were included: patients with BD, UR, and HC. All participants underwent The Schedules of Clinical Assessment in Neuropsychiatry (SCAN) interview (Wing et al. 1990) and a diagnosis of BD (or the lack of) was provided according to the International Classification of Diseases 10th version ICD-10 (WHO 1992).

All participants were assessed at baseline and every year for up to three years. Patients with BD were contacted every third month to identify new ongoing affective episodes. If the patients were in a new ongoing affective episode at the time of contact, they were scheduled for a new appointment with researchers on the BIO-team.

### Study participants

Patients with BD: Patients with newly diagnosed BD living in the Capital Region of Denmark are offered a two-year program at the Copenhagen Affective Disorder Clinic Copenhagen, Denmark (Kessing et al. 2013). Inclusion criteria were newly diagnosis of BD or newly diagnosis of a single manic episode according to the ICD-10 and an age of 15–70 years.

Unaffected relatives: Unaffected first-degree relatives, siblings or children, to the patients included in the BIO-study, were recruited after permission from patients with BD. Exclusion criteria were any previous or current psychiatric diagnosis lower than F34.0 according to ICD-10 (i.e., organic mental disorders, mental and behavioral disorders due to psychoactive substance use including alcohol, schizophrenia or other psychotic disorders, affective disorders).

Healthy control individuals: Healthy control persons were recruited among blood donors, age 15–70, from the Blood Bank at Rigshospitalet, Copenhagen. Exclusion criteria were treatment requiring psychiatric disorder in the individual or one of the individuals’ first-degree family members.

At all visits, three observer-based rating scales and one self-reported questionnaire were administered in addition to daily smartphone-based self-reported and automatically generated activity measures.

### Observer-based ratings of activity

In all three groups, the severity of depressive and manic symptoms for the past three days was clinically evaluated using the Hamilton Depression Rating Scale 17-items (HAMD) (Hamilton 1967) and the Young Mania Rating Scale (YMRS) (Young et al. 1978), respectively. On the HAMD-17 we used sub-item 8 addressing psychomotor retardation and sub-item 9 addressing psychomotor agitation. We assumed that an observer-based rating of psychomotor retardation and agitation to some degree reflects activity and energy level. If psychomotor retardation/agitation is rated high, we assume that patients have more difficulties being both physically and socially active. Therefore, we used this sub-item. On the YMRS we used sub-item 2 evaluating the level of motor activity and sub-item 6 addressing the pressure of speech. These items were specifically chosen to investigate whether smartphone-based self-reported and automatically generated activity measurements reflect these clinically assessed activity measurements. The Functional Assessment Short Test (FAST) was included to investigate if changes in daily smartphone-based self-reported or automatically generated activity are reflected in changes in functioning, as assessed by clinical researchers. The Functional Assessment Short Test is specifically developed for bipolar disorder and addresses six areas of functioning for the past 14 days: autonomy, occupational functioning, cognitive functioning, financial issues, interpersonal relationship and leisure time. All items are rated from 0 (no difficulties) to 5 (severe difficulties). The test has a high test–retest reliability and has been validated against the Global Assessment of Functioning scale (GAF) (Rosa et al. 2007).

### Self-reported physical activity questionnaire

Self-reported physical activity level was assessed by using The International Physical Activity Questionnaires – short form (IPAQ). IPAQ is a widely used questionnaire to address the level of physical activity and sedentary behavior (Lee et al. 2011).

The IPAQ provides information regarding time spent in four intensity levels: (1) vigorous-intensive activity, (2) moderate-intensity activity, (3) walking and (4) sedentary for the past seven days (Craig et al. 2003). Summary measurements of overall self-reported physical activity are reported as a continuous variable metabolic equivalent task (MET minutes a week), representing the energy expended during the physical activity. Higher scores correspond to higher activity. The questionnaire was included to investigate whether smartphone-based self-reported and automatically generated activity is associated with patient-rated physical activity.

### Smartphone-based monitoring

All participants downloaded a smartphone-based app, Monsenso, on their smartphones. The Monsenso system consists of an app, where participants can self-monitor symptoms, and a web-based interface allowing clinicians and researchers to access participant's self-reported data (Bardram et al. 2013). The Monsenso app can be downloaded on both iPhone and Android smartphones and daily self-monitoring of symptoms were accessible on both iPhone and Android; however, in the present study automatically generated smartphone-based data were only accessible from participants using Android smartphones. Participants used their phones. Participants with no smartphone or participants having an iPhone were offered the opportunity to borrow an Android smartphone (LG Nexus 5) and use it as their primary phone during the study. The Monsenso system has a daily reminder function and self-reported data can be entered retrospectively for up to two days. Unaffected relatives and HC were asked to provide self-reported activity level daily for at least one month and preferably three months. Patients with BD were asked to report daily activity level for a minimum of three months. The BIO study is a comprehensive study and smartphone-based monitoring is only one part of the study.

### Smartphone-based activity measurements

In DSM-V a core symptom of bipolar disorder is changes in activity/energy level. Currently, there is no consensus regarding the definition and measurements of activity. In this study, we have investigated whether smartphone-based self-reported and automatically-generated activity measures can be used to monitor activity levels in patients with BD. We have not differentiated between physical and non-physical activity.

In the Monsenso app the three groups (patients with BD, UR, and HC) scored their daily activity level on a 7-point scale (−3, −2, −1, 0, 1, 2, 3). The daily activity level refers to the patient’s overall activity level for the day, it could refer to social—as well as physical activity, goal-directed activity, hyperactivity or another aspect of activity defined by the participant. For patients with BD daily mood symptoms were collected on a 9-point scale from depressed to manic (−3, −2, −1, −0.5, 0, 0.5, 1, 2, 3). Self-reported neutral mood was defined as a self-reported mood score of −0.5, 0, or 0.5. In the present study, a limited number of automatically generated attributes were available including (1) number of steps; (2) incoming- and outgoing text messages; (3) call duration and number of incoming-, outgoing- and missed calls; (4) seconds the screen is on (referred to as screen time) and number of times the screen is turned on. Of these attributes, we hypothesized that step counts to most likely reflect physical activity and the other attributes most likely to reflect social activity.

### Statistical methods

All hypothesis and statistical analyses were planned á priori. We investigated smartphone-based activity measurements against: (1) IPAQ addressing physical activity for the past week; (2) subitem 8 and 9 HAMD-17 and subitem 2 and 6 on YMRS, addressing items that are related to activity and energy level for the past three days; and (3) FAST, addressing functioning the past 2 weeks. In these analyses, we calculated averages of smartphone-based activity measurements reported in the period corresponding to the days assessed by the questionnaires and rating scales. Therefore, only visits where participants had provided data on self-reported activity corresponding to the days of the questionnaire were included. All participants were included in the analyses.

Secondly, we validated daily smartphone-based self-reported activity against the automatically generated activity measurements. In these analyses we only used days where participants provided both self-reported and automatically generated data. Thirdly, differences in activity measurements between the three groups were investigated. The following activity measurements were included: self-reported smartphone-based activity, automatically generated smartphone-based activity, physical activity (IPAQ), activity assessed according to activity sub-items on HAMD and YMRS, respectively, and FAST. Smartphone-based self-reported and automatically generated activity collected during the whole study period were used regardless of affective episodes. The participants were assessed annually and additionally, patients with BD were booked for a new appointment with a researcher from the BIO-team if they were experiencing a new ongoing affective episode. Therefore, some participants provided repeated measurements of clinical assessed activity.

Linear mixed-effect models were used in all analyses. This model can account for participant-specific correlations by including familial relationship and participants id number as random effects. In analyses comparing the difference in activity between the patients with BD, UR and HC groups were used as a fixed effect. For each comparison, we considered an unadjusted model and a model adjusted for age and sex. The model accounts for unbalanced data and allows us to use all data points from each study participant during follow-up and not only complete datasets. Thus, one of the advantages of the linear mixed model (LMM) analysis is that it implicitly imputes missing data from dropouts under the assumption that these are *missing at random*. As such, handling missing data is embedded in the LMM procedure.

Model control was performed for each analysis. Prior studies investigating smartphone-based self-reported and automatically generated activity are scarce and no standard measurements were accessible. Thus, due to the explorative nature of the study adjustment for multiple testing was not done and p-values < 0.05 (two-tailed) were considered statistically significant. All analyses were conducted using the Statistical Package of the Social Sciences (SPSS) Version 22.

### Ethical considerations

The Bipolar Illness Onset (BIO) study has been approved by the ethics committee in the Capital Region, Copenhagen, Denmark (ref. nr. H-7-2014-007) and the Danish Data Protection Agency, Capital Region of Copenhagen (protocol no.: RHP-2015-023). The study was conducted in accordance with the Declaration of Helsinki and all participants provided written informed consent.

First degree relatives (UR) were compensated with a gift card equivalent of 40 USD, whereas patients with BD and HC participants were not compensated. The UR were compensated to enhance recruitment of participants to the study.

## Results

### Socio-demographic and clinical characteristics

In the study period from September 2016 to February 2019, 240 patients with BD, 66 UR and 118 HC were included in the BIO-study cohort. Self-reported data on activity measured via smartphones were collected from 203 patients with BD, 54 UR, and 109 HC. Primary reasons for non-participation were time, surveillance concerns or that the participant had no smartphone and did not want to borrow a smartphone. Participants possessing an Android smartphone additionally provided automatically generated smartphone-based data (75 patients with BD, 15 UR, and 32 HC).

Socio-demographic and clinical characteristics are presented in Table 1. Among the patients with BD there was no statistically significant difference between patients who provided smartphone-based recordings (203 patients) and those who did not (37 patients with BD) and between patients who had an iPhone vs. Android smartphone with regard to age, sex, educational level, or illness duration (*p’s* > 0.5).

**Table 1:** Socio-demographic and clinical characteristics of patients with bipolar disorder (BD), unaffected first-degree relatives (UR) and healthy control individuals (HC) at baseline

	BD	UR	HC	BD vs HC (*p)*	BD vs UR (*p)*	UR vs HC (*p)*
Participants, n	203	54	109			
Age, years	28 [24–35]	26 [22–31]	26 [24–36]	0.39	0.028	0.014
Female sex, % (n)	69.0 (140)	56.6 (30)	63.3 (69)	0.31	0.064	0.34
Education, years	15 [13–17]	15 [13–17]	16 [15–17]	<0.001	0.27	0.09
Full time employment, % (n)	32.0 (64)	48.1 (25)	50.0 (52)	0.002	0.03	0.82
Student, % (n)	40.5 (81)	46.2 (24)	38.5 (40)	0.73	0.46	0.36
HAMD-17	9 [5–15]	2 [0–3]	0 [0–2]	<0.001	<0.001	0.60
YMRS	2 [0–7]	0 [0–2]	0 [0–1]	<0.001	<0.001	0.60
FAST, total score	22 [13–30]	1 [0–3]	0 [0–2]	<0.001	<0.001	0.20
Bipolar disorder type II, % (n)	66.5 (135)	–	–	–	–	–
Age of onset, years	17 [14–21]	–	–	–	–	–
Illness duration, years^a^	10 [5–14]	–	–	–	–	–
Untreated BD, years^b^	4 [1–10]	–	–	–	–	–
No. prior depressive episodes	5 [3–13]	–	–	–	–	–
No. prior hypomanic episodes	5 [2–15]	–	–	–	–	–
No. prior manic episodes	1 [1–2]	–	–	–	–	–
No. prior mixed episodes	1 [1–2]	–	–	–	–	–
No. prior total episodes	12 [6–27]					
Episode at baseline^f^						
Full or partiel remisison, % (n)	60.6 (123)					
Hypomania/mania, % (n)	5.9 (12)					
Depression, % (n)	33.0 (67)					
Mixed, % (n)	0.5 (1)					

Continuous variables are presented as median [interquartile range] and *p-*values are calculated based on differences in mean between the tree groups using mixed models. Categorical data are presented as % (n) and *p*-values are calculated by using the chi-square test.

*HAMD-17* Hamilton Depression Rating Scale with 17 items version, *YMRS* Young Mania Rating Scale, *FAST* Functional Assessment Short Test.

^a^Illness duration was defined as the time from the first episode to the time of inclusion.

^b^Untreated BD was defined as time from the first mania, hypomania, or mixed episode to time of diagnosis. Episode at baseline according to HAMD-17 and YMRS, full or partial remission was defined as HAMD-17 and YMRS < 14, scores on HAMD-17 or YMRS ≥ 14 were defined as depression or hypomania/mania and as mixed if both HAMD-17 and YMRS ≥ 14;

During the study period, participants provided a total of 48,747 daily self-reported smartphone-based activity ratings. The self-reported activity ratings measured via smartphones were provided for a median of 106 days [interquartile range (IQR): 48–204] for patients with BD, 81 days [IQR: 35–121] for UR, and 82 days [IQR: 40–121] for HC. Automatically generated smartphone-based data were collected for a total of 29,879 days with a median of 216 days [IQR: 76–375] for patients with BD, 163 days [IQR: 107–427] for UR, and 214 days [IQR: 92–393] for HC. All UR and HC and 93% of patients with BD provided above one month of automatically generated smartphone-based data. Self-reported data were provided above one month for 80% of participants. The patients with BD were seen annually and upon development of a new mood episode. In this study, the patients with BD contributed with 337 visits. For the majority of visits, patients were in full or partial remission (65%) (HAMD and YMRS < 14), 27% had a HAMD score ≥ 14 and 7% had a YMRS score ≥ 14.

### Daily self-reported activity via smartphones

The upper part of Table 2 presents associations between daily self-reported smartphone-based activity and self-reported physical activity according to scores on the IPAQ, functioning according to scores on the FAST and activity items on the HAMD and YMRS rating scales. Daily self-reported smartphone-based activity was statistically significantly associated with all validity measurements, except in relation to item 6 on the YMRS rating scale (speech).

**Table 2:** Associations between smartphone-based activity measurements^a^ and self-reported physical activity and observer-based rating scales for depression, mania and functioning (data pooled for patients with bipolar disorder, unaffected relatives and healthy control individuals)

	Model 1			Model 2		
	B	95% CI	*p*	B	95% CI	*p*
Self-reported smartphone-based activity N = 482						
IPAQ	1.17	0.80;1.54	* < 0.001*	1.16	0.79;1.53	* < 0.001*
HAMD-17 sub-item 8	−0.44	−0.58:−0.29	* < 0.001*	−0.45	−0.60;−0.31	* < 0.001*
HAMD-17 sub-item 9	−0.33	−0.50;−0.16	* < 0.001*	−0.35	−0.52;−0.17	* < 0.001*
YMRS sub-item 2	0.22	0.09;0.36	*0.002*	0.23	0.09;0.37	*0.001*
YMRS sub-item 6	0.08	−0.02;0.17	0.10	0.08	−0.02;0.17	0.11
FAST	−0.020	−0.026;−0.014	* < 0.001*	−0.20	−0.26;−0.15	* < 0.001*
Step counts						
IPAQ N = 74	0.02	−0.32;0.36	0.90	0.007	−0.34;0.36	0.97
HAMD-17 sub-item 8 N = 88	−750.5	−1800;299.8	0.16	−668.4	−1749;412.3	0.22
HAMD-17 sub-item 9	−617.9	−1932;696.1	0.35	−643.7	−1967;679.7	0.34
YMRS sub-item 2	134.1	−997.9;1266	0.81	85.2	−1055;1226	0.88
YMRS sub-item 6	−187.8	−945.4;569.8	0.62	−192.5	−970.6;585.6	0.62
FAST N = 97	−40.9	−73.5;−8.28	*0.015*	−39.1	−72.1;−6.04	*0.021*
Screen time (seconds/day) N = 179						
IPAQ N = 116	−0.67	−1.35;0.01	0.053	−0.57	−1.25;0.11	0.10
HAMD-17 sub-item 8 N = 137	2603	1064;4141	*0.001*	2177	−612.5;3741	*0.007*
HAMD-17 sub-item 9	2658	422.0;4893	*0.020*	2488	286.2.4690	*0.027*
YMRS sub-item 2	−701.0	−2589;1187	0.46	−600.4	−2457;1256	0.52
YMRS sub-item 6	531.8	−755.1;1818	0.42	566.5	−700.1;1833	0.38
FAST N = 145	111.7	36.1;187.4	*0.004*	92.2	17.3;167.0	*0.016*
Screen on (number/day) N = 179						
IPAQ N = 116	−0.17	−2.77;2.44	0.90	0.51	−2.16;3.17	0.71
HAMD-17 sub-item 8 N = 137	−10.5	−18.0;−3.06	*0.006*	−11.8	−19.2;−4.33	*0.002*
HAMD-17 sub-item 9	−9.29	−20.3;1.76	0.098	−10.1	−21.1;0.79	0.069
YMRS sub-item 2	8.96	−0.17;18.1	0.054	8.72	−0.34;17.8	0.059
YMRS sub-item 6	2.92	−3.26;9.10	0.35	3.53	−2.62:9.68	0.26
FAST n = 145	−0.29	−0.64;−0.06	*0.11*	−0.32	−0.67;−0.03	*0.071*
Incoming calls (number/day) N = 180						
IPAQ N = 137	−0.04	−0.74;0.82	0.92	−0.01	−0.08;0.08	0.98
HAMD-17 sub-item 8 N = 155	0.13	−0.12;0.40	0.31	0.14	−0.11;0.39	0.28
HAMD-17 sub-item 9	0.40	0.07;0.74	*0.019*	0.41	0.07;0.74	*0.019*
YMRS sub-item 2	0.15	−0.12;0.41	0.28	0.14	−0.13;0.40	0.32
YMRS sub-item 6	0.14	−0.02;0.30	0.087	0.14	−0.02;0.31	0.086
FAST N = 167	0.005	−0.004;0.015	0.31	0.006	−0.004;0.016	0.26
Outgoing calls (number/day) N = 180						
IPAQ N = 137	0.68	−0.80;0.22	0.36	−0.66	−0.86:0.22	0.39
HAMD-17 sub-item 8 N = 155	−0.13	−0.61;0.36	0.61	−0.12	−0.61;0.38	0.63
HAMD-17 sub-item 9	0.35	−0.31;1.02	0.30	0.37	−0.30;1.03	0.28
YMRS sub−item 2	0.95	0.46;1.45	* < 0.001*	0.94	0.43;1.44	* < 0.001*
YMRS sub-item 6	0.27	−0.05;0.58	0.097	0.27	−0.05;0.59	0.092
FAST N = 167	−0.009	−0.029;0.012	0.42	−0.007	−0.027;0.014	0.52
Missed calls (number/day) = 180						
IPAQ N = 137	−0.08	−0.60;0.44	0.78	−0.10	−0.64;0.43	0.70
HAMD-17 sub-item 8 N = 155	0.13	−0.06;0.31	0.17	0.13	−0.05;0.32	0.16
HAMD-17 sub-item 9	0.002	−0.25;0.25	0.99	0.007	−0.25;0.26	0.96
YMRS sub-item 2	0.03	−0.17;0.23	0.75	0.04	−0.16;0.25	0.70
YMRS sub-item 6	0.04	−0.08;0.16	0.54	0.03	−0.08;0.16	0.58
FAST N = 167	0.005	−0.020;0.011	0.17	0.005	−0.002;0.011	0.18
Duration of phone calls (seconds/day) N = 180						
IPAQ N = 137	−0.01	−0.07;0.05	0.74	−0.01	−0.07;0.06	0.79
HAMD-17 sub-item 8 N = 155	−2.77	−216;210	0.98	−15.3	−230;199	0.89
HAMD-17 sub-item 9	91.2	−199.1:381.5	0.54	69.5	−219.6;358.6	0.64
YMRS sub-item 2	202,5	−24,3;429,3	0.080	208.1	−18.9;435.1	0.072
YMRS sub-item 6	74.5	−65.4;214.3	0.29	72.6	−66.9;212.1	0.43
FAST N = 167	0.27	−5.34;9.88	0.56	1.89	−5.78;9.58	0.63
Incoming text messages (number/day) N = 180						
IPAQ N = 133	0.17	−0.19;0.52	0.35	0.11	−0.25:0.46	0.55
HAMD-17 sub-item 8 N = 159	0.54	−0.62;1.71	0.36	0.67	−0.49;1.82	0.26
HAMD-17 sub-item 9	1.00	−0.57;2.56	0.21	1.12	−0.43;2.67	0.16
YMRS sub-item 2	−0.50	−1.78;0.78	0.38	−0.46	−1.73;0.81	0.48
YMRS sub-item 6	0.18	−0.64;0.99	0.67	−0.09	−0.71;0.89	0.83
FAST N = 168	−0.005	−0.046;0.044	0.98	−0.002	−0.047;0.042	0.92
Outgoing text messages (number/day) N = 180						
IPAQ N = 133	0.26	−0.85;0.60	0.14	0.19	−0.16,0.53	0.29
HAMD-17 sub-item 8 N = 159	0.29	−0.82;1.93	0.61	0.38	−0.72;1.47	0.49
HAMD-17 sub-item 9	0.71	−0.77;2.20	0.34	0.81	−0.65;2.27	0.23
YMRS sub-item 2	−0.56	−1.77;0.64	0.36	−0.51	−1.70;0.68	0.40
YMRS sub-item 6	0.06	−0.70;0.82	0.87	−0.01	−0.77;0.74	0.97
FAST N = 168	−0.02	−0.06;0.03	0.43	−0.02	−0.06;0.02	0.38

Significant *p* values are given in italic

*IPAQ* The Physical Activity Questionnaire—short form, a measure of physical activity 7 days prior to assessment, *HAMD-17* The Hamilton Depression Rating Scale 17-item. Sub-item 8 and 9 addressing psychomotor retardation and agitation, respectively, *YMRS total* The Young Mania Rating Scale. Subitem 2 and 6 addressing motor activity and speech, respectively, *FAST* The Functional Assessment Short Test, a measure of global functioning 14 days prior to assessment

^a^Smartphone-based activity measurements: Averages of smartphone-based activity ratings were calculated for the current day and 3 days before ratings with HAMD-17 and YMRS, 7 days prior for rating IPAQ and 14 days prior for FAST.

### Daily automatically generated smartphone-based activity measurements

The remaining part of Table 2 presents similar associations for automatically generated smartphone-based activity. None of the automatically generated smartphone-based activity features were associated with scores on IPAQ. Step count and screen time were associated with FAST. The number of outgoing calls was associated with item 2 on the YMRS rating scale, the number of incoming calls was positively associated with item 9 on HAMD rating scale, number of times the screen was turned on was negatively associated with item 8 on HAMD and screen time was positively associated with both item 8 and 9 on HAMD. The rest of the automatically generated smartphone-based activity measurements were not associated with clinically validated activity measurements.

### Daily self-reported smartphone-based activity versus automatically generated smartphone-based activity measurements

As can be seen from Table 3, daily self-reported activity via smartphone was statistically significantly associated with all measurements of smartphone-based activity, except missed calls and incoming calls, which were borderline statistically significant.

**Table 3:** Associations between self-reported^a^ and automatically generated^b^ smartphone-based data for all participants in the study owning an Android smartphone

	Model 1			Model 2		
	B	95% CI	*p*	B	95% CI	*p*
Self-reported smartphone-based activity						
Step count (number/day)	0.37	0.30;0.43	* < 0.001*	0.37	0.30;0.44	* < 0.001*
Screen time (seconds/day)	−0.13	−0.15;−0.11	* < 0.001*	−0.13	−0.15;−0.11	* < 0.001*
Screen on (number/day)	0.005	0.004;0.005	* < 0.001*	0.005	0.004;0.005	* < 0.001*
Call duration (seconds/day)	−0.20	−0.34;−0.07	*0.004*	−0.20	−0.34;−0.07	*0.004*
Incoming calls (number/day)	0.01	−0.001;0.027	0.063	0.013	−0.001;0.027	0.064
Outgoing calls (number/day)	0.03	0.02;0.04	* < 0.001*	0.030	0.02;0.04	* < 0.001*
Missed calls (number/day)	0.015	−0.001;0.030	0.064	0.015	−0.001;0.030	0.063
Incoming text-messages (number/day)	0.006	0.003;0.009	* < 0.001*	0.006	0.003;0.009	* < 0.001*
Outgoing text-messages (number/day)	0.005	0.003;0.007	* < 0.001*	0.005	0.003;0.007	* < 0.001*

Significant *p* values are given in italic

*Model 1* unadjusted, *Model 2* adjusted for age and sex.

^a^Self-reported smartphone-based activity rated on a scale from −3 to + 3.

^b^Automatically generated smartphone-based activity features reflecting social parameters and physical activity

### Daily smartphone-based self-reported and automatically generated activity measurements in patients with newly diagnosed BD, UR, and HC.

**Fig. 1:**
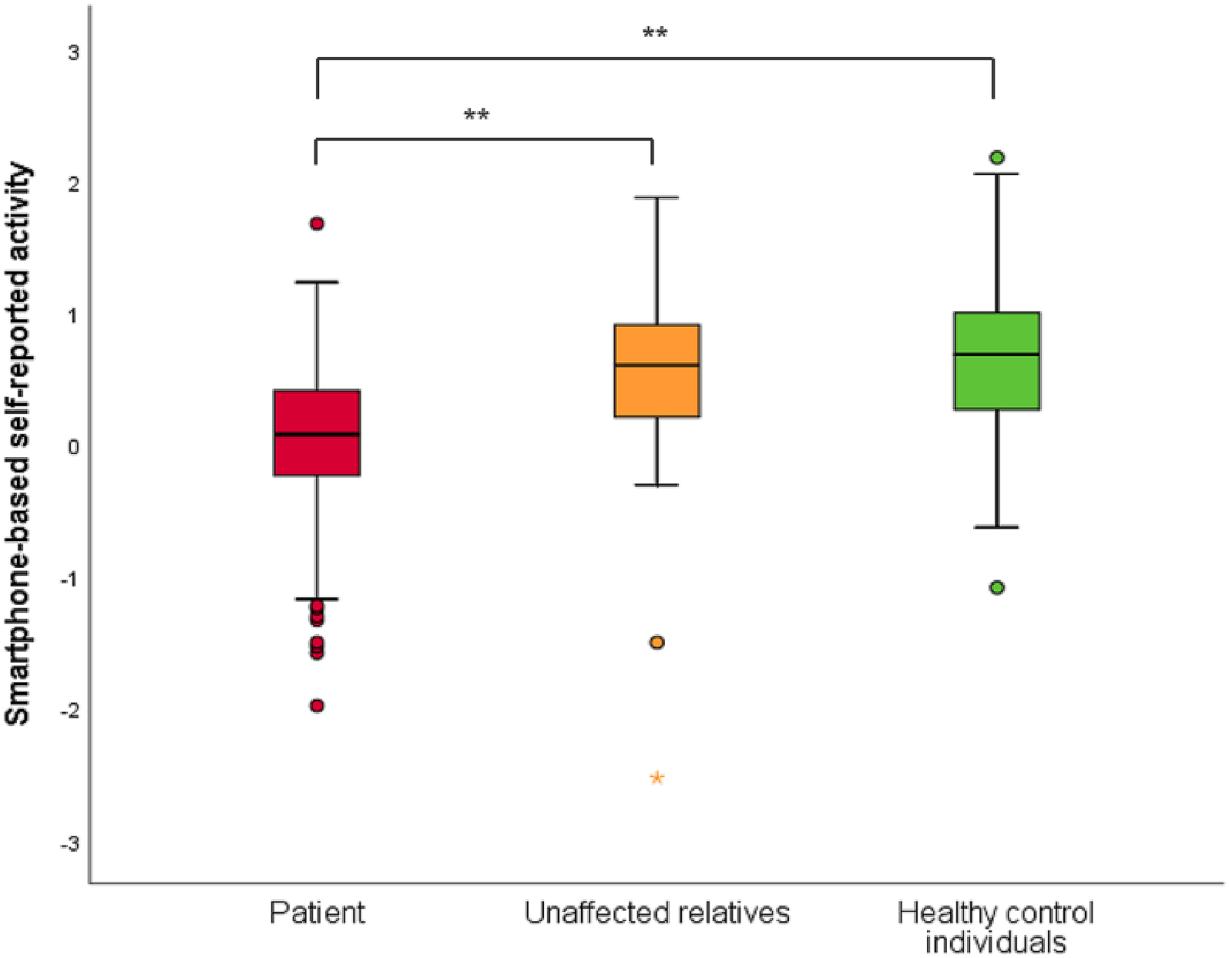
Boxplots of smartphone-based self-reported activity in patients with newly diagnosed bipolar disorder, unaffected first-degree relatives and healthy control individuals. The bottom and top of the box represent the first and third quartiles and the upper and lower whiskers extend from the box to the largest and lower value, respectively. No further than 1.5 times the interquartile range from the box. Data beyond the whiskers are plotted individually. **p < 0.001

As can be seen from Fig. 1 and the upper part of Table 4, patients with BD had a statistically significantly lower mean level of daily self-reported mean activity level and fewer days with high activity and more days with low activity compared with HC. In sub-analysis, where only days at which patients had self-reported remitted mood (−0.5 to 0.5 on the mood scale) were included, patients with BD also had statistically significantly lower mean activity levels compared with UR and HC. Unaffected relatives did not differ from HC individuals on any measure of self-reported activity.

**Table 4:** Estimated differences in activity in patients with bipolar disorder (BD), unaffected first-degree relatives (UR) and healthy control individuals (HC)

	Model 1									Model 2								
	BD		UR		HC		BD/HC	BD/UR	UR/HC	BD		UR		HC		BD/HC	BD/UR	UR/HC
	Mean	95% CI	Mean	95% CI	Mean	95% CI	*p*	*p*	*p*	Mean	95% CI	Mean	95% CI	Mean	95% CI	*p*	*p*	*p*
Smartphone-based self-reported activity																		
Self-reported activity	0.06	−0.02;0.14	0.54	0.39;0.69	0.68	0.57;0.79	* < 0.001*	* < 0.001*	0.16	0.05	−0.03;0.14	0.56	0.40;0.72	0.68	0.57;0.79	* < 0.001*	*0.001*	0.25
High activity level^a^ (%)	12.5	10.4;14.7	24.2	20.0;28.4	24.8	21.9;27.8	* < 0.001*	* < 0.001*	0.81	12.7	10.4;15.0	24.3	20.1;28.6	24.9	21.9;27.9	* < 0.001*	* < 0.001*	0.82
Low activity level^b^ (%)	14.0	12.2;15.7	8.0	4.6;11.4	5.1	2.7;7.5	* < 0.001*	*0.002*	0.18	14.4	12.6;16.3	7.8	4.4;11.2	5.5	3.1;8.0	* < 0.001*	*0.001*	0.29
Automatically generated smartphone-based activity																		
Step counts (number/day)	3502	2711;4292	3177	1456;4896	5911	4511;7310	*0.004*	0.73	*0.016*	3622	2774;4470	2851	1018;4684	5923	4506;7340	*0.006*	0.45	*0.010*
Screen time (min/day)	205.4	179;232	188.8	130;247	175.1	135;215	0.22	0.61	0.70	197.3	170;224	188.7	130;247	176,4	137;216	0.39	0.79	0.73
Screen on (number/day)	66.8	56.6;77.1	82.3	59.7;104.9	71.1	55.6;86.6	0.65	0.22	0.42	63.9	53.6;74.1	77.9	55.8;100.1	70.8	55.8;85.8	0.45	0.26	0.60
Incoming calls (number/day)	1.50	1.30;1.71	1.11	0.68;1.53	1.39	1.08;1.71	0.57	0.09	0.27	1.56	1.35;1.76	1.67	0.73;1.61	1.40	1.09;1.71	0.40	0.10	0.38
Outgoing calls (number/day)	2.69	2.33;3.05	2.28	1.50;3.06	2.37	1.83;2.91	0.33	0.35	0.86	2.74	2.38;3.10	2.47	1.70;3.23	2.39	1.87;2.91	0.28	0.53	0.87
Missed calls (number/day)	1.00	0.89;1.12	0.77	0.56;0.98	0.75	0.56;0.93	*0.023*	*0.042*	0.86	0.99	0.87;1.10	0.82	0.64;1.00	0.76	0.57;0.94	*0.036*	*0.045*	0.62
Call duration (min/day)	17.8	15.0;20.7	9.93	3.70;16.2	10.45	6.17;14.73	*0.005*	*0.024*	0.89	17.8	14.9;20.6	11.15	4.93;17.4	10.7	6.45;14.9	*0.007*	0.058	0.90
SMS incoming (number/day)	6.56	5.50;7.63	6.27	3.92;8.62	5.22	3.61;6.84	0.17	0.82	0.47	6.63	5.53;7.73	6.49	4.11;8.88	5.25	3.63;6.87	0.17	0.92	0.39
SMS outgoing (number/day)	5.25	4.19;6.31	5.45	3.12;7.79	4.25	2.64;5.86	0.30	0.88	0.40	5.26	4.17;6.35	5.76	3.40;8.12	4.30	2.69;5.90	0.33	0.70	0.31
Physical activity (IPAQ)																		
IPAQ total score	2748	2414;3080	3049	2421;3676	3374	2953;3795	*0.022*	0.41	0.40	2752	2410;3095	3045	2412;3679	3303	2877;3729	*0.045*	0.42	0.51
Observer-based rating scales																		
HAMD subitem 8	0.40	0.34;0.46	0.10	−0.02;0.21	0.01	−0.02;0.21	* < 0.001*	* < 0.001*	0.20	0.39	0.33;0.45	0.08	−0.04;0.19	−0.003	−0.10;0.08	* < 0.001*	* < 0.001*	0.30
HAMD subitem 9	0.34	0.28;0.40	0.12	0.01;0.23	0.01	−0.07;0.09	* < 0.001*	* < 0.001*	0.11	0.36	0.30:0.41	0.08	−0.02:0.19	0.02	−0.05:0.10	* < 0.001*	* < 0.001*	0.40
YMRS subitem 2	0.41	0.33;0.49	0.24	0.09;0.39	0.14	0.04;0.25	* < 0.001*	0.04	0.30	0.42	0.34;0.50	0.24	0.09;0.39	0.16	0.06;0.27	* < 0.001*	0.033	0.42
YMRS subitem 6	0.62	0.52;0.73	0.08	−0.12;0.28	0.05	−0.09;0.19	* < 0.001*	* < 0.001*	0.81	0.64	0.53;0.74	0.07	−0.14;0.27	0.07	−0.08;0.21	* < 0.001*	* < 0.001*	0.96
FAST	20.8	19.5;22.0	3.25	0.76;5.6	1.38	−0.33;3.09	* < 0.001*	* < 0.001*	0.22	20.4	19.1;21.7	2.70	0.21;5.19	1.15	−0.59;2.89	* < 0.001*	* < 0.001*	0.32

Significant *p* values are given in italic

*Model 1* unadjusted, *Model 2* adjusted for age and sex, *Physical activity (IPAQ)* The Physical Activity Questionnaire short form. A measure of physical activity 7 days prior to assessment calculated as MET minutes per week, *HAMD-17* The Hamilton Depression Rating Scale 17-item. Subitem 8 and 9 addressing psychomotor retardation and agitation, respectively, *YMRS total* The Young Mania Rating Scale. Subitem 2 and 6 assessing motor activity and speech, respectively, *FAST* The Functional Assessment Short Test (FAST), a measure of global functioning 14 days prior to assessment at baseline.

^a^High activity level: Calculated as proportion of days with activity level >  = 2 on a scale from −3 to + 3/number of days with activity ratings.

^b^Low activity level: Calculated as proportion of days with activity level <  = −2 on a scale from −3 to + 3/number of days with activity ratings.

The midpart of Table 4 shows that patients with BD had statistically and significantly lower number of steps, more missed calls and longer duration of calls per day compared with HC individuals. Unaffected relatives had a lower number of steps per day compared with HC individuals. There were no statistically significant differences in any other automatically generated smartphone-based measure of activity*.*

## Discussion

This study is the first to systematically validate smartphone-based activity measurements in bipolar disorder and to compare both daily self-reported and automatically generated smartphone-based activity among patients with BD, UR, and HC individuals. Overall, we confirmed our three hypotheses. Firstly, smartphone-based self-reported activity was a valid measure according to scores on the IPAQ and activity items on the HAMD and YMRS, and was associated with FAST scores, whereas automatically generated smartphone-based activity measurements were weakly correlated with these measurements. Secondly, daily self-reported smartphone-based activity measurements and automatically generated smartphone-based activity measurements correlated with all measurements (except missed calls and incoming calls that were borderline statistically significant). Thirdly, patients with BD had decreased daily self-reported activity compared with HC, and UR did not differ from HC individuals. According to automatically generated smartphone-based data, patients with BD had decreased physical (number of steps) and social activity (more missed calls) but a longer call duration compared with HC, whereas UR had decreased physical activity, only.

### Self-reported and automatically generated smartphone-based activity

Changes in activity level is a central feature in patients with BD. However, no clear consensus concerning the definition or assessment of the term activity exists (Scott et al. 2017). Several terms have been used to describe different aspects of activity (e.g. hyperactivity, goal-directed activity, behavioral activation) (Scott et al. 2017). These terms reflect changes in both psychomotor activities, body movement, and behavior (Lewinsohn and Graf 1973). Also, there is no consensus regarding how “activity” can or should be measured. Notably, we found that daily self-reported smartphone-based activity was associated with clinically assessed measurements of energy/activity, psychomotor retardation and agitation, and functioning in addition to all measurements of automatically generated smartphone-based activity (except missed calls and incoming calls, which were borderline statistically significant). This result shows the advantages of remotely monitoring. Remotely reported activity levels are a new area of research in bipolar disorder. We are aware of one other study on remotely reported activity, only, finding associations, although weak, between remotely self-reported energy level and activity items on validated questionnaires (Tsanas et al. 2016). Other studies that have investigated self-reported activity have used the term “energy” to evaluate activity/energy levels (Tsanas et al. 2016; Abdullah et al. 2016). Although we in this study, investigated self-reported daily activity reflecting overall activity as defined by the participant, it is likely that self-reported energy and self-reported activity reflect two different aspects of activity/energy, and it would be interesting to investigate these aspects further.

Unexpectedly, in the present study, the associations between automatically generated smartphone-based data and the clinical measurements of activity (sub-items on YMRS and HAMD-17) were not as compelling as hypothesized. An explanation for this discrepancy may be that all of our automatically generated smartphone-based activity attributes were investigated separately. Future studies should consider integrating several smartphone-based features including both self-reported data and automatically generated data and apply machine learning methods to develop a composite marker to estimate overall activity level. A composite marker reflecting overall activity may have clinical utility in both diagnosis and treatment monitoring of bipolar disorder (Abdullah et al. 2016).

In contrast, the participants' daily self-reported activity via smartphone was associated with all measurements of automatically generated smartphone-based activity (except missed calls and incoming calls, which were borderline statistically significant). This finding is in accordance with results from a few other studies reporting an association between remotely collected self-reported energy in patients with BD and automatically generated smartphone-based data (Abdullah et al. 2016) and between remotely collected self-reported energy and motor activity measured by actigraphy (Merikangas et al. 2018).

### Differences in activity level between patients with bipolar disorder, unaffected relatives and healthy control individuals

A lower mean level of activity has been reported during remission in patients with BD compared with HC (Crescenzo et al. 2017; Scott et al. 2017) and first-degree relatives, respectively (Pagani et al. 2016). In line with this, we found a lower mean level of self-reported smartphone-based activity in patients with BD compared with HC. Other studies investigating remotely reported activity level either have a small sample size (Schwartz et al. 2016) or have not reported findings regarding differences in self-reported activity between groups (Tsanas et al. 2016). Decreased activity level has previously been associated with mood level (Merikangas et al. 2018) and related to affective episodes (Rosa et al. 2010). Moreover, reduced engagement in activities may be predictive of forthcoming depressions (Weinstock and Miller 2008).

Recently, our group published a study presenting automatically generated smartphone-based data as a potential diagnostic behavioral marker for BD, also discriminating patients with BD during euthymia from healthy control individuals (Faurholt-Jepsen et al. 2019). In the present study, step counts, missed calls and call duration differed between patients with BD and HC substantiating the validity of these physical and social activity measurements. Remarkably, the number of incoming calls and text messages did not differ between the three groups, which might reflect that patients in the study who were newly diagnosed with BD have a normal social network. A recent review found inconsistency in the association between text-messages and affective states and concluded that text-logs should be interpreted with caution due to competing communication platforms (Rohani et al. 2018). Nevertheless, the design and measurements in studies investigating automatically generated smartphone-data are highly heterogenous and with small sample sizes, which might explain inconsistencies across studies.

## Limitations

First, automatically generated smartphone-data allow us to collect data on behavioral activities unobtrusively and could have potential as a valid state marker and possible trait marker for bipolar disorder (Faurholt-jepsen et al. 2018). However, in this study, only a few of the automatically generated smartphone-based data differed between the three groups. An explanation could be that smartphone-based automatically generated data could only be collected from participants with Android smartphones, which resulted in a low number of participants, especially UR. Therefore, results should be interpreted with caution and negative findings could be due to type II errors.

Second, we have included all data in the analysis, regardless of the patient with BD’s mood state and therefore, we cannot capture mood state-dependent variations in activity level. Assessment of such changes will be possible during the longitudinal part of this study were the sample size is larger and patients have provided smartphone-based recordings for a longer period. Third, all participants included in this study were also a part of the larger BIO study. In a study solely investigating smartphone-based monitoring, the level of adherence may have been higher. Fourth, participants used their own phones. Consequently, data were gathered from multiple different platforms, which might cause some heterogeneity in data not accounted for in this study. Also, the collection of automatically generated smartphone-based data may be highly influenced by the time period the data were collected. During the past ten years, there have been changes in the use of text messages due to emergence of alternative communications platforms such as social media, Snapchat etc. and the technology software used by smartphones have advanced considerably (Alhabash and Ma 2017). To obtain a comprehensive understanding of the putative relationship between smartphone-based activity and a participant’s general activity, future studies may need to address person-specific variations in phone usage and the transient popularity of commercial communication applications. In this study, we accounted for some of this variation by adjusting for age and sex. Fifth, there are a number of limitations in using step counts to address physical activity. Participants can carry their phone in different places (pocket, handbag, jacket, etc.) and are likely not to use the phone during some physical activities as swimming or cycling). Therefore, a better estimate for physical activity would be to combine step counts with other attributes such as self-reported activity, location data and/or collect data from a wearable device. Sixth, other automatically generated features such as accelerometer, ambient light and microphone could have provided useful information. However, these parameters are battery consuming and to enhance long-term adherence to the smartphone-application these parameters were not included. Seventh, participants agreeing to participate in this study might represent a sub-population with a more technology-friendly approach increasing the risk of selection bias. Further, our healthy control group were recruited among blood donors and might represent a “super-healthy” control group. Eightly, two-thirds of the patients included in the study had a BD type II and two-thirds were female and therefore findings may not be generalized to all patients with BD.

## Strengths

First, the study comprised of 366 systematically recruited participants including patients with newly diagnosed BD with a median age of 28 years and their URs. Additionally, patients with BD were diagnosed at a specialized mood disorder clinic and diagnosis (and lack of diagnosis) was verified for all participants with a SCAN-interview conducted by trained assessors. Secondly, we used clinically validated activity measurements from the HAMD-17, YMRS, and FAST rating scales. Thirdly, the Monsenso system used in the study is well validated and importantly fulfilling safety of data storage and privacy requirements.

## Conclusion

Daily self-reported smartphone-based activity measurements represent a valid marker for overall activity and correlate with measurements of automatically generated smartphone-based activity. Daily collected smartphone data of activity differs between individuals with BD, UR, and HC. The study suggests that daily self-reported smartphone-based activity measurements and some automatically generated smartphone-based activity measurements represent clinical meaningful markers that may be clinically useful in diagnosis and treatment monitoring of bipolar disorder.

## Data Availability

The study is ongoing; therefore, the research data are not shared.

## Abbreviations

BD: Bipolar Disorder
BIO-study: The Bipolar Illness onset study
DSM-5: Diagnostic and Statistical Manual of Mental Disorders, Fifth Edition
FAST: Functional Assessment Short Test
GAF: Global Assessment of Functioning scale
HAMD: Hamilton Depression Rating Scale
HC: Healthy Control Individuals
ICD-10: International Statistical Classification of Diseases and Related Health Problems 10^th^ Revision
IPAQ: International Physical Activity Questionnaire
IQR: Interquartile range
MET minutes: Metabolic Equivalent Task minutes
UR: Unaffected relatives
YMRS: Young Mania Rating Scale
